# Whey Protein Hydrolysates of Sheep/Goat Origin Produced by the Action of Trypsin without pH Control: Degree of Hydrolysis, Antihypertensive Potential and Antioxidant Activities

**DOI:** 10.3390/foods11142103

**Published:** 2022-07-15

**Authors:** Lambros Sakkas, Eleni Lekaki, Golfo Moatsou

**Affiliations:** Laboratory of Dairy Research, Department of Food Science and Human Nutrition, Agricultural University of Athens, Iera Odos 75, 11855 Athens, Greece; lasakkas@hotmail.com (L.S.); lekakie2@gmail.com (E.L.)

**Keywords:** WPH, sheep/goat whey WPC, trypsin hydrolysis, degree of hydrolysis, ACE inhibitory activity, DPPH radical scavenging activity, iron chelating activity

## Abstract

Tryptic WPHs with considerable residual whey protein content intact were developed from two sheep/goat WPCs (65% and 80% protein) without pH control. Pasteurization was used to avoid denaturation. Changes in non-protein nitrogen (DH_TCASN), free amino groups (DH_TNBS), and major whey proteins were used to investigate the degree and extent of hydrolysis. Antihypertensive potential (ACE-IA), radical scavenging (DPPH-RSA), and iron chelation (Fe-CA) were assessed. No statistically significant changes in pH (5.84–6.29) were observed during hydrolysis and storage. At the start of hydrolysis, DH_TCASN was ≅11% for both substrates whereas DH_TNBS was >10% and >5% for WP65 and WP80, respectively. After one-hour hydrolysis, DH_TCASN was ≅17% for both substrates and DH_TNBS was ≅15% and ≅11% for WP65 and WP80, respectively. The β-lactoglobulin, α-lactalbumin, and caseinomacropeptide of WP65 were hydrolyzed by 14 ± 1.3%, 73.9 ± 2.6% and 37 ± 2.6%. The respective values for WP80 were 14.9 ± 1.7%, 79.9 ± 1%, and 32.7 ± 4.8%. ACE-IA of the hydrolysates of both substrates was much higher (>80%) than that of controls (<10%). Hydrolysis, substrate type, and storage did not affect the DPPH-RSA (45–54%). Fe-CA of the WP65 and WP80 hydrolysates were ≅40% and ≅20%, respectively; a similar outcome was found in the respective controls. Refrigerated storage for 17 h did not affect the degree of hydrolysis and biofunctional activities.

## 1. Introduction

Whey proteins (WP) exhibit an array of valuable technological and nutritional properties, which are transferred to a variable extent into different types of whey powders and fractions. WP have convenient gelation and emulsification behaviors, interact with other macromolecules, exhibit foaming and thickening properties, water holding capacity, and are both biocompatible and biodegradable. Moreover, edible films, coatings, hydrogels, and nanoparticles encapsulating active food and drug components can be manufactured from the whey fraction of milk [1,2]. Functionality refers to the behavior of WP under heat treatments, the ability to stabilize emulsions and foams acting as interfacial films, the formation of edible films, and the ability to produce elastic gel networks [3]. Whey protein concentrate (WPC) and whey protein isolate (WPI) powders are the result of the selective fractionation of whey by removing fat and a significant amount of lactose, minerals, and non-protein nitrogen compounds; therefore, they contain a high amount of the major whey proteins of milk mostly found in the native form. There are numerous applications for this category of products, such as in different food systems, infant and nutraceutical formulae, biomedical products, biodiesel, bioplastics, and textiles [4]. Several studies have shown the health effects of WP and their peptides, i.e., antioxidant, immunomodulatory and anticancer potential, regulation of diabetes and factors of cardiovascular disease, and treatment of liver disease and sarcopenia [2].

Various types of modifications and treatments either used alone or in combination are applied for the improvement of the physicochemical, functional, nutritional, and biological properties of whey and WP products, i.e., heat treatments to induce controlled polymerization, ultrasound, high-pressure, pulsed-electric field or membrane processing, radiation treatment, chemical modifications—mainly the formation of glycol-conjugates via Maillard reaction—and enzymatic treatments such as cross-linking by transglutaminase or hydrolysis by proteases of animal or microbial origin. Treatment with proteases results in the production of whey protein hydrolysates (WPH) that refer to both experimental and commercial products [3,5,6].

The outcome of hydrolysis is related to the enzyme type, enzyme to substrate (E/S) ratio, temperature, pH, and time. The structure of WP, the mechanism and kinetics of the enzymatic hydrolysis, the enzymes utilized for hydrolysis, the peptides derived therefrom, and the effect of the pre-treatment of substrates have been reviewed by Cheison and Kulozik [5]. Conditions of proteolysis that are adequate for the production of whey-derived biofunctional peptides, as well as their purification and characteristics, have been presented by Dullius et al. [7]. In contrast to the numerus reports on the WPH of cow milk origin, the publications on the properties of WPH originating from small ruminants’ milk are very limited and concern the hydrolysis of non-fractionated whey or individual WP. Indicatively, in-vitro inhibitory activity of human platelet aggregation of tryptic hydrolysates of sheep and goat caseinomacropeptide (CMP) was studied [8,9]. The angiotensin converting enzyme inhibitory activity (ACE-IA) of tryptic [10] or other types of hydrolysates [11] of sheep and goat β-lactoglobulin (β-LG) has been assessed. The antioxidant and ACE-IA of sheep cheese whey hydrolyzed by bacterial protease has been presented [12]. Commercial goat milk was hydrolyzed by subtilisin and trypsin to optimize the conditions resulting in the maximum degree of hydrolysis and ACE-IA [13]; later, the same hydrolysis scheme was used to develop artificial neuronal networks for modelling hydrolysis [14]. Recently, the suitability of various proteases for the production of goat milk WPHs regarding their emulsifying ability and hypoglycemic potential has been investigated [15].

WPCs manufactured from sheep/goat Feta cheese whey are differentiated from that of cow counterparts due to high β-LG content [16]. The usual ratio of sheep to goat milk in Feta cheese milk is 70:30. WPHs from this type of WPC have been used for the supplementation of yoghurt-type gels [17,18]. Tryptic WPHs using these types of WPC as substrate were the objective of the present study. The aim of these experiments was the development of WPHs with high residual intact native protein content. For this purpose, hydrolysis by trypsin was performed under non-controlled pH conditions to avoid the dilution of the hydrolysate and the increase in ash content-. Moreover, low pasteurization was applied to stop the hydrolysis and avoid the denaturation of intact major whey proteins. The assessment of the hydrolysis outcome was carried out by the degree of proteolysis and the investigation of the biofunctional potential.

## 2. Materials and Methods

### 2.1. Substrates, Hydrolysis and Sampling

Two types of whey protein concentrates powders (WPCs) with approximately 65% (WP65) and 80% (WP80) total protein content of sheep/goat origin manufactured from Feta cheese whey were used as substrates. The WPC powders were reconstituted in distilled water at a concentration of 5%, *w*/*w*. After stirring for 60 min, the WPC solutions were stored at 4–6 °C overnight for proper hydration. Each solution was divided in two parts: the first was used as hydrolysis substrate while the other was a non-hydrolyzed control that was treated similarly in terms of heating and storage.

Hydrolysis was performed by means of bovine trypsin (TPCK treated, T1426, Sigma-Aldrich, St. Louis, MO, USA) at a 0.25% enzyme to protein ratio. Incubation was carried out for one hour at 50 °C without pH adjustment at pH 6.1–6.3 that was the pH of WPC dilutions. The reaction was stopped by heat treatment at 68 °C for 10 min, i.e., typical milk low-pasteurization conditions, to avoid denaturation of whey proteins. Experiments were performed in triplicate.

Samples of the hydrolysates and their respective controls were taken just after the addition of trypsin, at 0.05 h (t0.05), after one h of hydrolysis and subsequent heat inactivation (t1) and after 17 h stay of t1 samples at refrigerated storage (t18). Control samples for each experiment were the respective WPC dilutions without enzyme addition treated under the same conditions. Sample codes are presented in Table 1.

### 2.2. Analyses

#### 2.2.1. Protein Composition of the Substrates

Total nitrogen (TN), nitrogen soluble at pH 4.6 (SN), and nitrogen soluble at 12% trichloroacetic acid (TCASN) of the WPC powders (substrates) were determined by the Kjeldahl method. Total protein (TP) was estimated as total nitrogen (TN) × 6.38. Nitrogen fractions were prepared as follows: the pH of substate solutions was adjusted at pH 4.6 using 1 M HCl; after centrifugation at 3000× *g* for 30 min, the supernatants were filtrated using a Whatman No 41 filter paper and the filtrate was the SN fraction; equal quantities of SN and 24% (*w*/*w*) trichloroacetic acid (TCA) were mixed and remained at room temperature for two hours; the precipitate was removed using Whatman No 40 filter paper and the filtrate was the TCASN fraction; the concentration of the major native whey proteins of the substrates was estimated by reversed-phase HPLC (RP-HPLC) using a Vydac C4 214 TP 515 column [14]; the major whey proteins of the profiles were the caseinomacropeptide (CMP), α-lactalbumin (α-LA), and β-lactoglobulin (β-LG).

#### 2.2.2. Degree and Extent of Hydrolysis

The degree of hydrolysis (DH) was assessed by:

i. The quantitative changes of nitrogen soluble at pH 4.6 (SN) and nitrogen soluble at 12% trichloroacetic acid (TCASN), as described in Section 2.2.1. The degree of hydrolysis (DH) based on SN changes was estimated according to DH_SN (%) = 100 × (SN_HYD-SN_CON)/(TN_CON-TCASN_CON), where HYD is a hydrolysate and CON is the respective control. The expression TN_CON-TCASN_CON corresponds to protein N content. The DH based on TCASN changes (DH_TCASN) was estimated accordingly.
ii. Changes of free amino groups was determined by the TNBS (picrylsulfonic acid solution, P2297, Sigma Aldrich, St. Louis, MO, USA) method of Adler-Nissen [19], with some modifications. One hundred μL hydrolysate was diluted with 900 μL of 1% SDS (sodium dodecyl sulfate); 250 μL of this dilution were diluted further with 2 mL 0.21 M phosphate buffer, pH 8.0. After the addition of 2 mL 0.05%, *w*/*w* TNBS, the mixture was incubated in the dark at 50 °C for 60 min. The reaction was stopped by the addition of 4 mL 0.1 M HCl. After cooling down for 30 min, the absorbance at 340 nm was determined. Glycine was used for the construction of the standard curve. The estimation of the DH was according to Spellman et al. [20]: DH_TNBS (%) = 100 × [(AN2-AN1)/123.3]. AN1 is the amino acid nitrogen content of the control WPC solution without trypsin (CON) expressed as mg/g protein and was estimated using the reference curve. Accordingly, AN2 is the content of the respective hydrolysate (HYD). The value 123.3 mg/g is the nitrogen content of the peptide bonds of the whey proteins in the substrate.

The extent of hydrolysis (EH) was assessed by the reduction of the concentration of major native whey proteins, β-LG, α-LA, and CMP, using the RP-HPLC method (Section 2.2.1). The control solution or hydrolysate was diluted with 0.1% trifluoracetic acid in ultrapure water at 1:3 ratio, centrifuged at 10,000 × *g* for 10 min, and the supernatant was clarified with a PVDF 0.45 μm syringe filter before injection.

#### 2.2.3. Biofunctionality Assays

The antihypertensive and antioxidant potential of the hydrolysates were assessed as previously described [21] with some modifications.

Angiotensin converting enzyme inhibitory activity (ACE-IA) was determined by the RP-HPLC method [19]. The hydrolysates after one hour of hydrolysis and after refrigerated storage and the respective controls were diluted in ultrapure water at a ratio 1:2. The dilution was centrifuged at 3000× *g* for 15 min and filtered with a PVDF 0.45 μm syringe filter before injection.

For the antioxidant potential, assays for DPPH radical scavenging activity (DPPH-RSA) and Fe^2+^ chelating activity (Fe-CA) after 30 and 60 min of reaction were performed [19]. The DPPH-RSA reaction mixture consisted of 200 μL hydrolysate or control, 720 μL methanol, and 80 μL of 1 mM DPPH solution in methanol. For the Fe-CA assay, the hydrolysates or controls were diluted with ultrapure water at a 1:50 ratio and the dilution was centrifuged at 9500× *g* for 10 min. The supernatant was assayed after filtration.

#### 2.2.4. Statistical Analysis

Analysis of variance (ANOVA) was used to study the effect of hydrolysis time and storage of the substrate on hydrolysis indices, as well as the results of biofunctionality assays. Differences were investigated by means of least significant difference test (LSD, *p* < 0.05).

## 3. Results and Discussion

### 3.1. Degree and Extent of Hydrolysis

The protein profile and the nitrogenous fractions of the WPC powders used as substrates are shown in Table 2. The contribution of SN fraction to the TN of WP80 was lower compared to WP65, which indicates that processing conditions applied to increase the degree of concentration induced partial denaturation of whey proteins. The high level of protein concentration of the former induced partial denaturation or structural changes of whey proteins that made them insoluble at pH 4.6.

The release of H^+^ from the hydrolysis of peptide bonds decreases the pH of the hydrolysates [19]. The changes of pH during hydrolysis and storage presented in Table 3 were not statistically significant. As reported in the literature, the lack of pH control in hydrolyses performed using papain or a microbial alternative [22], alcalase [23], or commercial enzyme preparations [24] did not affect the degree of hydrolysis.

**Figure 1:**
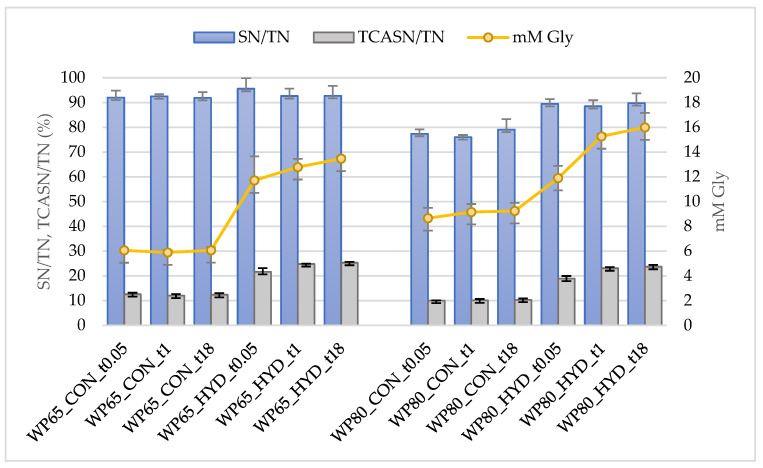
Proteolysis indices of tryptic hydrolysates of 5% (*w*/*w*) solutions of WPC powders of sheep/goat origin manufactured from Feta cheese whey in comparison to non-treated control counterparts. Abbreviations are explained in Table 1 and Section 2.2.1. Means and standard deviations of three experiments.

**Figure 2:**
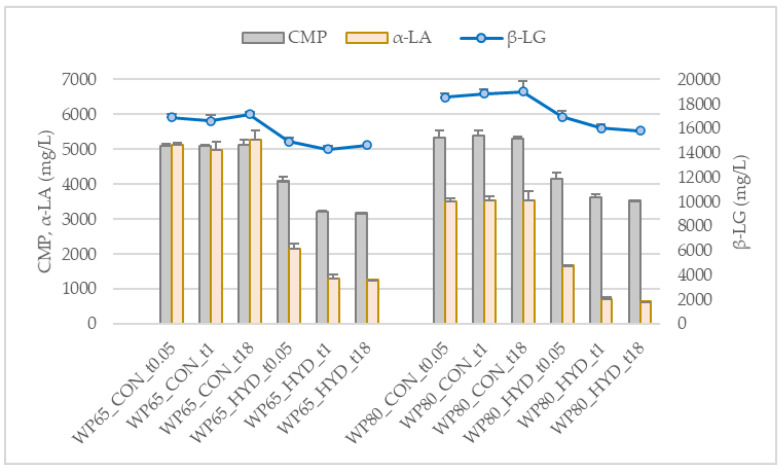
Concentration of the major intact whey proteins in the tryptic hydrolysates of 5% (*w*/*w*) solutions of WPC powders of sheep/goat origin manufactured from Feta cheese whey in comparison to non-treated control counterparts. Abbreviations are explained in Table 1 and Section 2.2.1. Means and standard deviations of three experiments.

Figure 1 presents the evolution of the concentration of pH 4.6 soluble nitrogen (SN), 12% TCA soluble nitrogen (TCASN), and the free amino groups estimated by the TNBS method (mM Gly). The control samples (CON) and the respective hydrolysates (HYD) were analyzed at the start (t0.05) and end of hydrolysis (t1) and after refrigerated storage (t18). The concentration of major individual native WP under the same conditions is presented in Figure 2.

From both Figure 1 and Figure 2, it is evident that the nitrogen and protein profiles of control (CON) solutions of WPCs without enzyme were not affected by time and storage under refrigeration. As expected, insignificant changes of the soluble nitrogen (SN) of WP65 substrate after hydrolysis took place due to lack of casein in this category of powders. However, an increase in SN was observed in the hydrolysate of WP80, which could be assigned to the trypsin action on the non-native whey proteins of the pH 4.6-insoluble fraction of WP80 that produced soluble peptides. (Table 2). Hydrolysis changed the composition of the SN fraction of both substrates by increasing the medium- and small-sized peptides that remain soluble at 12% TCA (TCASN); the same is true for the free amino groups (mM Gly). The specificity of trypsin on lysine bonds could explain the strong absorbance of the hydrolysates in the TNBS method. Lysine at the end of the produced peptides reacts intensively with TNBS due to the existence of two primary amino groups [18].

Table 4 presents different expressions utilized to assess the outcome of hydrolysis in relation to non-hydrolyzed controls (Section 2.2.2). The degree of hydrolysis (DH) was estimated by the quantitative changes of soluble nitrogen fractions (DH_SN and DH_TCASN) and of free amino groups (DH_TNBS). The extent of hydrolysis (EH) was calculated using the changes of the major residual native whey proteins (EH_β-LG, EH_α-LA and EH_CMP). In accordance with Figure 1, time did not cause any statistically significant change of the DH_SN (*p* > 0.05) of both substrates. It was very limited for WP65 substrate ranging on average within 0.8% and 1.1% opposite of the 13.7–15.3% estimated for WP80. In general, hydrolysis indices of Table 4 exhibited substantial changes within the first minutes of hydrolysis.

Some of the hydrolysis indices estimated in the present study (Section 2.2.2 and Table 4) were differentiated between the WP65 and WP80. DH_SN and EH_α-LA of WP80 hydrolysates were significantly higher (*p* < 0.05) than that of WP65 counterparts. The opposite was true for DH_TNBS. In particular, DH_TCASN was approximately 11% for both substrates, whereas the DH_TNBS was more than 10% and 5% for WP65 and WP80, respectively. The main part of the increase in DH within the first minutes was due to the reduction of native α-LA by 57.6% and of CMP by 19.9%. The linear correlation coefficients between the hydrolysis indices shown in Table 5 were statistically significant (*p* < 0.05). Strong correlation coefficients were observed between DH_TCASN and EH_α-LA and EH_CMP while the DH_TCASN and DH_TNBS were moderately correlated.

According to the present results (Table 4), the extent of hydrolysis of CMP in WP65 and WP80 was similar: one-hour hydrolysis caused a statistically significant decrease (*p* < 0.05) in the intact CMP. CMP is the 106–169 N-terminal hydrophilic part of κ-casein. Lys111–Lys112, Lys112–Asn113, and Lys116–Thr117 are the trypsin susceptible bonds of cow CMP. Enzyme and substrate concentration and hydrolysis mode—batch or continuous—control whether all three bonds are hydrolyzed [25]. These particular bonds exist in sheep and goat CMPs [26,27]. Reported peptides corresponding to susceptible bonds in the tryptic digest of sheep CMP are f106–112, 112–116, 112–123, and 117–123; among them, the CMP f112–116 exhibits a high platelet aggregation inhibition [8]. These peptides have been also found in the tryptic digests of both sheep and goat CMPs [9].

At the end of one-hour hydrolysis, there was a statistically significant (*p* < 0.05) further increase in DH_TCASN and decrease in α-LA and CMP concentrations following the significant increase in DH_TNBS (Table 4). In the majority of studies, β-LG is hydrolyzed more intensively than α-LA by trypsin under experimental conditions close to optima pH 8 and 37 °C [28,29]. An exception is the study of Ferreira et al. [30], which was performed in 5% WPC80 solution at constant pH 8.0 and 37 °C and reports a high residual concentration of non-hydrolyzed β-LG. After 15, 30, and 60 min approximately 87%, 65%, and 42% of the initial remained intact, as opposed to the almost complete hydrolysis of α-LA. Similar to our findings, enhancement of α-LA hydrolysis by trypsin has been observed under non-controlled pH “free-fall pH” conditions [31]. Moreover, trypsin preferably hydrolyzed α-LA further than β-LG during the keeping of a WPI hydrolysate under pH 5.5–6.5 [32].

Trypsin exhibits narrow specificity for the N-terminal side of Arg and Lys residues in the protein [5]. The trypsin cleavage sites of β-LG of small ruminants’ milk do not differ from that of cow milk [33], which is the most studied kind of milk. The characteristics of WPHs—apart from the enzyme—are affected by several factors, such as: pH, temperature and time of hydrolysis, enzyme to substrate ratio, and total solids [5,34]. The high concentration of intact β-LG contrast to a high extent of cleavage α-LA can be attributed to the conditions of hydrolysis at non-controlled pH close to pH 6.0 at 50 °C and to the particular protein composition of the substrate. The pH conditions affect both the activity of the enzyme and the structure and solubility of major whey proteins. At milk pH 6.6, β-LG is present as a dimer—depending on temperature—below 5.5 and tends to form octamers while at pH > 7.5 occurs as a monomer [35,36]. The main part of relevant studies have been performed under the optimum temperature 37 °C and pH 7.8 for trypsin, where β-LG occurs as a monomer that facilitates the action of trypsin. Temperature and pH of hydrolysis affect the stability and solubility of both the enzyme and whey proteins. At 55 °C, trypsin activity is quickly decreased and, between pH 6 and pH 4.25, the enzyme is slowly denatured losing its activity at pH < 4.25 [5]. The isoelectric point of β-LG is close to pH 5.2 and that of α-LA ranges within pH 4.2–4.5 [36]. Therefore, reduced solubility of β-LG under the pH conditions of the present experiments (Table 3) could prevent the access of the enzyme in contrast to α-LA. The decrease in pH slows down and orders the hydrolysis of β-LG due to a resistance of the core domain to trypsin without affecting the release of N- and C-terminal fragments [37]. At pH 6.0, fewer fragments are detected compared to pH 7.0 because of the effect of pH on trypsin stability and activity [37]. Taking the aforementioned points into account, the more intense hydrolysis of α-LA compared to β-LG found in the present study can be explained.

A large part of the changes in indices in Table 4 were observed soon after the addition of trypsin (t0.05) and remained steady after 17 h of refrigerated storage (t18). The fast hydrolysis can be assigned to the high hydrolysis temperature [37,38]. At pH 7.5 at 50 °C and after 10 min, the degree of hydrolysis of β-LG by trypsin with E/S ratio of 0.05 has been found similar to that at 35 °C at 20 min. The acceleration of hydrolysis is assigned to a higher initial activity at temperatures higher than the optimum 37 °C; however, at 50 °C, fewer peptides were produced compared to the hydrolysates incubated at 30–45 °C [38]. Moreover, a temperature of 50 °C at pH 7 has been suggested as a second optimum for short reaction times producing a less controlled hydrolysis pattern since both the α-LA and β-LG are similarly attacked [28]. Cheison and Kulozik [5] concluded that the first few minutes are critical for the outcome of hydrolysis.

The higher β-LG concentration compared to α-LA of the WPCs of the present study could also contribute to the deviation of hydrolysis pattern from that of other reports. The variation of substrate concentration under a constant E/S ratio affects the outcome of hydrolysis and suggests that the formed peptides can inhibit the enzyme [39]. In addition, the processing conditions used in WPC production could limit the access of trypsin to cleavage sites on β-LG. According to a previous study [16], pasteurization of whey, ultrafiltration, evaporation, and spray-drying decrease the percentage of native on the total protein content in these types of WPCs. Heat induced modifications such as oxidation and glycation of proteins, formation of lysine-alanine crosslinks, pH, the exposure of cleavage sites due to protein unfolding, and the structure of aggregates affect the hydrolysis and digestibility of whey proteins [40]. The modified lysine residues of β-LG lactosylated under mild heat treatments has been found resistant to trypsin [41]. Accordingly, glycated β-LG is reported to be resistant to trypsin/chymotrypsin digestion in contrast to its unglycated counterpart [42]. When non-native β-LG monomers were used as substrate, the accessibility of trypsin to cleavage sites was substantially reduced but the release of functional peptides was enhanced [43]. However, the structural changes of β-LG induced by mild or moderate heating are affected by the environment of WPC that contains other whey proteins and variable quantities of lactose and minerals [44].

### 3.2. Biofunctional Potential

#### 3.2.1. Peptidyl Dipeptide Hydrolase-Inhibitory Activity (ACE-IA)

**Figure 3:**
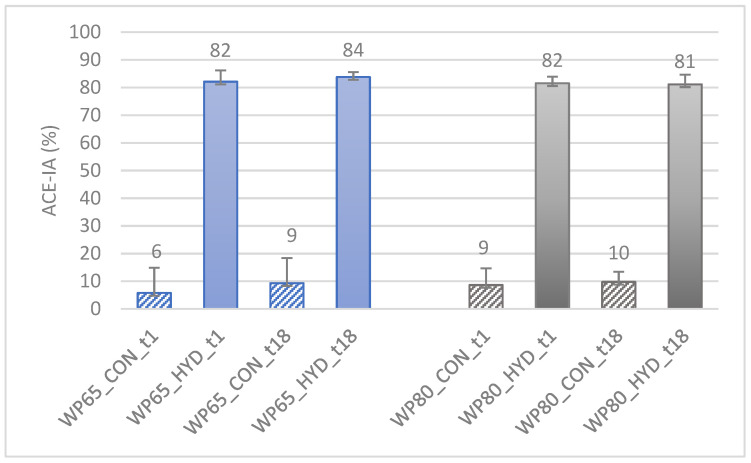
ACE-IA (%) of the tryptic hydrolysates of 5% (*w*/*w*) solutions of WPC powders of sheep/goat origin manufactured from Feta cheese whey in comparison to non-treated counterparts. Abbreviations explained in Table 1 and Section 2.2.3. Means and standard deviations of three experiments.

Under the conditions of our experiments, one hour of hydrolysis of both substrates resulted in hydrolysates with high ACE-IA—more than 80%—that remained steady during refrigerated storage (Figure 3). The type of substrate—WP65 or WP80—did not influence the outcome. Under the same conditions, the non-hydrolyzed substrates exhibited an ACE-IA lower than 10%.

A high ACE inhibition index has been estimated for the tryptic hydrolysates of cow α-LA, β-LG, and WPC80 [45]. ACE-IA is related to the existence of hydrophobic amino acid residues at the C-terminal end and a molecular mass less than 3000; further degradation of tryptic peptides may strengthen or reduce their ACE-inhibitory potential [5,45]. The most common features of ACE inhibiting antihypertensive peptides derived from β-LG and α-LA, i.e., lactokinins, have been concisely reviewed [7]. They consist of 2–12 amino acids, have a molecular mass lower than 1000 Da, and aromatic AA at the C-end, while Leu, Ile, Val, Lys, and Arg are important for ACE inhibition. No relationship between ACE and DH obtained by different enzymes is reported because the specificity of the enzymes determines the production of ACE inhibitory peptides [46,47]. Trypsin produces hydrolysates from WP with high ACE-IA, such as cow β-LG (f22–25, 32–40, 81–83) and of cow α-LA (f104–108, 99–108) [48]. ACE inhibitory peptides have been also isolated from the tryptic hydrolysates of whey proteins of small ruminants’ milk, e.g., β-LG (f1–8, 142–148) [10] and CMP (f106–111, 106–112) [49]. The release of ACE inhibitory peptides in the tryptic hydrolysates of β-LG is affected by heat-denaturation of the substrate and the pH conditions [43]. Production of peptides from the N-terminal region is reduced, especially at pH 5.1, because the conformational changes occurring in this part of the protein are involved in the association of denatured WP. At pH 5.1, the production of ACE-inhibitory peptides from the C-terminal region of the denatured molecule is inhibited but the opposite is true for pH 6.8 and 8.0 [43].

#### 3.2.2. Antioxidant Activities

Antioxidant potential is related to different types of activities and their combinations, i.e., radical scavenging, inhibition of lipid peroxidation, or chelation of metal ions [12].

DPPH radical scavenging activity (DPPH-RSA) of the hydrolysates and of the respective controls is shown in Table 6, in comparison to that of the referenced antioxidant, Trolox. No statistically significant changes were observed between the controls and their hydrolysates, during the storage of hydrolysates and between the WP65 and WP80 hydrolysates.

**Figure 4:**
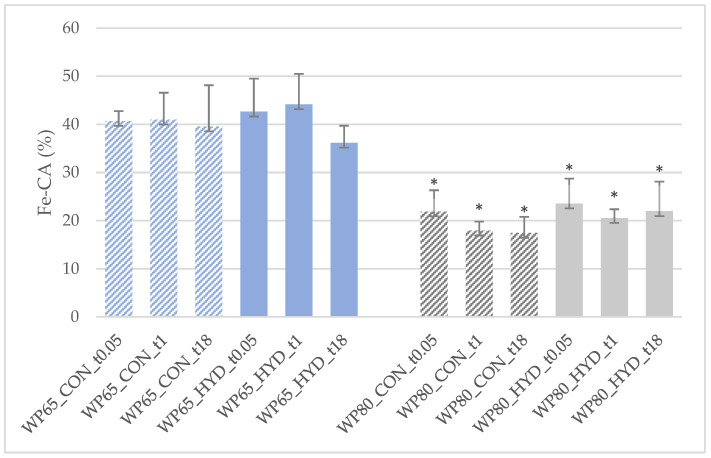
% Fe^2+^ chelating activity (Fe-CA) after a 30 min reaction of the tryptic hydrolysates of 5% (*w*/*w*) solutions of WPC powders of sheep/goat origin manufactured from Feta cheese whey in comparison to non-treated counterparts. 1 Abbreviations explained in Table 1 and Section 2.2.3. Means and standard deviations of three experiments. * Statistically significant differences (*p* < 0.05) between WP80 and WP65 counterparts.

The % Fe^2+^ chelating activity (Fe-CA) of the hydrolysates and their respective controls is presented in Figure 4. No statistically significant changes were observed between controls and their hydrolysates, during the storage. However, the hydrolysates of WP80 exhibited significantly lower (*p* < 0.05) Fe-CA compared to WP65 counterparts.

Among the antioxidative proteins of the whey fraction of milk that could tolerate the conditions of WPC manufacture are the lactoferrin, which can bind considerable amounts of the pro-oxidative iron ions, and the enzyme superoxide dismutase [50]. Radical absorbance capacity is related to the scavenging of free radicals by sulfur containing amino acids [51] to some peptides derived from whey proteins, mainly from α-LA and β-LG [52], and to the secondary structure of the peptides [53]. Fe-CA in milk and dairy products is attributed mainly to casein and casein-derived peptides and especially phosphopeptides, which can interact with metals via the polar side chains of some amino acids [52,53]. The antioxidant activity of peptides is affected by the methods used for the isolation of proteins-substrates, the DH, the type of enzyme, the peptide concentration, and the peptide structure, i.e., composition, configuration, and hydrophobicity [54]. Aromatic residues in the amino acids can exhibit radical scavenging properties by donating protons to electron deficient radicals. The imidazole group in histidine-containing peptides exhibit metal ion-chelating ability and the SH group of cysteine is a direct radical scavenger. Hydrophobic amino acids facilitate the accessibility to hydrophobic radicals and polyunsaturated fatty acids. Carboxyl and amino groups in the side chains can act as chelators of metal ions [54].

In contrast to our findings, an increase in DPPH-RSA has been reported in WPH produced by the hydrolysis of a 15% cow WPC solution with trypsin at a DH close to 10% [55]. On the other hand, fractions of commercial WPHs with low scavenging activity containing mostly large peptides at low concentrations exhibited strong binding of iron, whereas cysteine from α-LA and β- LG seemed to be oxidized after hydrolysis [56]. The action of proteases used for the production of WPHs could modify or reduce the antioxidant potential of intact whey proteins. Moreover, whey proteins can be oxidized during processing at particular residues involved in the protein unfolding [57]. The results shown in Figure 4 coincide with these findings. The WP80 with more pH 4.6 insoluble whey protein (Table 2) exhibited lower Fe-CA activity than the less concentrated WP65 counterpart.

The comparison with the literature data for the antioxidant properties of WPHs produced and assayed under different conditions is inadequate. As previously discussed, the non-controlled pH conditions of hydrolysis resulted in a different peptide profile of the tryptic WPH. Cow WPC hydrolysis by commercial food enzyme preparation without pH control resulted in WPH with statistically significantly lower in vitro and cellular antioxidant activities compared to pH-controlled counterparts [24]. Moreover, lower oxygen radical absorbance capacity was estimated in non pH-controlled WP hydrolysates produced by papain compared to those produced under pH controlled conditions [22].

## 4. Conclusions

The conditions of hydrolysis of the present study—without pH control, incubation at 50 °C, duration of one hour, and stopping by pasteurization—that were rather marginal for trypsin action resulted in a limited degree of hydrolysis and a considerable amount of intact native whey proteins. Hydrolysis increased the ACE inhibitory activity of the WPCs dramatically, to more than 80%, without downgrading their radical scavenging and iron chelating ability. Moreover, the hydrolysis and biofunctionality indices of the hydrolysates remained steady during overnight refrigerated storage, which is a finding that facilitates their further processing. We suggest that the short reaction time, mild heat treatment, absence of additives, and substrate originating from small ruminants’ cheese whey is an emerging approach for the generation of WPHs. Future research work can be designed based on the present findings.

## Figures and Tables

**Table 1:** Coding of samples taken from the tryptic hydrolysates (HYD) of whey protein concentrates (WPC) of sheep/goat origin manufactured from Feta cheese whey and of the respective controls (CON). WP65, WPC with 65% protein content; WP80, WPC with 80% protein content ^1^.

Sample Code ^2^	Trypsin (0.25% on Protein)	Time (h) ^3^	Heating (68 °C for 10 min) ^4^	Storage (h) ^5^
WP(65 or 80)_CON_t0.05	-	0.05	+	-
WP(65 or 80)_CON_t1	-	1	+	-
WP(65 or 80)_CON_t18	-	1	+	17
WP(65 or 80)_HYD_t0.05	+	0.05	+	-
WP(65 or 80)_HYD_t1	+	1	+	-
WP(65 or 80)_HYD_t18	+	1	+	17

^1^ At a concentration of 5%, *w*/*w*; ^2^ in total 36 samples were taken from three repetitive hydrolyses for each substrate; ^3^ hydrolysis time at 50 °C in hours; ^4^ low-pasteurization for enzyme inactivation after one-hour hydrolysis; ^5^ storage time after heating under refrigeration.

**Table 2:** Protein composition of substrates WPC powders of sheep/goat origin manufactured from Feta cheese whey. Codes and abbreviations are explained in Section 2.1 and Section 2.2.1 Means and standard deviations of three experiments.

WPC	TP g/100 g	SN/TN %	TCASN/TN %	CMP g/100 g	α-LA ^1^ g/100 g	β-LG ^1^ g/100 g
WP65	61.25 ± 1.98	91.2 ± 2.62	12.9 ± 1.73	10.1 ± 0.32	10.4 ± 0.45	33.0 ± 3.35
WP80	73.27 ± 2.18	76.5 ± 2.74	10.6 ± 1.90	10.3 ± 0.58	6.7 ± 0.33	36.64 ± 1.04

^1^ in the native form.

**Table 3:** pH changes during hydrolysis and storage of tryptic hydrolysates of 5% (*w*/*w*) solutions of WPC powders of sheep/goat origin manufactured from Feta cheese whey. Codes are explained in Section 2.1.

Time (h)	WP65		WP80	
	Control	+Trypsin	Control	+Trypsin
0.05 ^1^	6.28 ± 0.03	6.28 ± 0.03	6.11 ± 0.10	6.11 ± 0.09
0.5 ^1^	5.92 ± 0.08	5.85 ± 0.07	5.75 ± 0.07	5.65 ± 0.07
1 ^1,2^	6.07 ± 0.19	5.95 ± 0.19	5.88 ± 0.18	5.84 ± 0.24
18 ^3^	6.35 ± 0.07	6.29 ± 0.12	6.19 ± 0.10	6.17 ± 0.12

^1^ 50 °C; ^2^ stopping of the reaction; ^3^ storage under refrigeration after stopping of the reaction.

**Table 4:** Degree of hydrolysis (DH) and extent of hydrolysis (EH) estimated as described in Section 2.2.2. Abbreviations explained in Section 2.2.4 and Table 1. Means and standard deviations of three experiments.

Hydrolysis Stage	DH_TCASN	DH_TNBS	EH_β-lg	EH_α-LA	EH_CMP
WP65_HYD_t0.05	11 ± 2.4 ^a^	10.6 ± 1.8 ^a,^*	11.6 ± 2.3	57.6 ± 4.6 ^a^	19.9 ± 2.9 ^a^
WP65_HYD_t1	17.1 ± 0.3 ^b^	13.7 ± 1.5 ^b,^*	14 ± 1.3	73.9 ± 2.6 ^b^	37 ± 2.6 ^b^
WP65_HYD_t18	17± 1.2 ^b^	14.8 ± 0.3 ^b,^*	14.6 ± 3.7	75.4 ± 3.3 ^b^	37.8 ± 0.9 ^b^
WP80_HYD_t0.05	10.9 ± 1.2 ^a^	5.9 ± 0.9 ^a^	8.6 ± 2.9 ^a^	51.9 ± 6.7 ^a^	21.9 ± 3.7 ^a^
WP80_HYD_t1	16.7 ± 1 ^b^	10.3 ± 1.1 ^b^	14.9 ± 1.7 ^b^	79.9 ± 1 ^b,^*	32.7 ± 4.8 ^b^
WP80_HYD_t18	17.4 ± 0.5 ^b^	11.3 ± 2 ^b^	16.9 ± 1.4 ^b^	82 ± 1.3 ^b,^*	33.6 ± 0.3 ^b^

Different letters indicate statistically significant differences (LSD, *p* < 0.05) within each group of samples; * indicates statically significant differences between respective hydrolysates of WP65 and WP80.

**Table 5:** Correlation coefficients between the hydrolysis indices of Table 4. as Abbreviations explained in Section 2.2.2 and Table 4.

	DH_TCASN	DH_TNBS	ED_β-LG	ED_α-LA	ED_CMP
DH_TCASN		0.66	0.72	0.91	0.89
DH_TNBS	0.66		0.58	0.61	0.73
ED_β-LG	0.72	0.58		0.83	0.64
ED_α-LA	0.91	0.61	0.83		0.81
ED_CMP	0.89	0.73	0.64	0.81	

**Table 6:** % DPPH-RSA estimated as described in Section 2.2.3. Abbreviations explained in Table 1 and Section 2.2.3; s Means and standard deviations (sd) of three experiments.

	WP65_CON _t0.05	WP65_CON _t1	WP65_CON _t18	WP65_HYD _t0.05	WP65_HYD _t1	WP65_HYD _t18	Trolox
mean	50.77	48.92	46.01	49.67	47.21	45.21	94.92
sd	7.05	4.40	3.48	5.26	5.44	4.64	2.25
	WP80_CON _t0.05	WP80_CON _t1	WP80_CON _t18	WP80_HYD _t0.05	WP80_HYD _t1	WP80_HYD _t18	Trolox
mean	49.13	51.12	54.05	49.68	51.55	51.39	96.51
sd	5.63	8.77	2.36	7.22	6.75	3.66	0.57

## Data Availability

Data is presented in the manuscript.
